# Comparison of the forearm rotation restriction capacities of four upper-extremity immobilization methods: there is no difference between single and double sugar tong splinting

**DOI:** 10.1186/s13018-024-04772-2

**Published:** 2024-05-06

**Authors:** Ali Engin Dastan, Arman Vahabi, Erhan Coskunol, Kemal Aktuglu

**Affiliations:** 1 Department of Orthopedics and Traumatology, Division of Hand Surgery, Faculty of Medicine, Ege University, Izmir, 35100 Turkey; 2 Department of Orthopedics and Traumatology, Faculty of Medicine, Ege University, Izmir, 35100 Turkey

**Keywords:** Forearm rotation, Cast, Sugar tong splint

## Abstract

**Background:**

The aim of this study was to compare the effects of four different immobilization methods [single sugar tong splint (SSTS), double sugar tong splint (DSTS), short arm cast (SAC), and long arm cast (LAC)] commonly used for restricting forearm rotation in the upper extremity.

**Methods:**

Forty healthy volunteers were included in the study. Dominant extremities were used for measurements. Basal pronation and supination of the forearm were measured with a custom-made goniometer, and the total rotation arc was calculated without any immobilization. Next, the measurements were repeated with the SAC, LAC, SSTS and DSTS. Each measurement was compared to the baseline value, and the percentage of rotation restriction was calculated.

**Results:**

The most superior restriction rates were observed for the LAC (*p* = 0.00). No statistically significant difference was detected between the SSTS and DSTS in terms of the restriction of supination, pronation or the rotation arc (*p* values, 1.00, 0.18, and 0.50, respectively). Statistically significant differences were not detected between the SAC and the SSTS in any of the three parameters (*p* values, 0.25; 1.00; 1.00, respectively). When the SAC and DSTS were compared, while there was no significant difference between the two methods in pronation (*p* = 0.50), a statistically significant difference was detected in supination (*p* = 0.01) and in the total rotation arc (*p* = 0.03).

**Conclusion:**

The LAC provides superior results in restricting forearm rotation. The SAC and SSTS had similar effects on forearm rotation. The DSTS, which contains, in addition to the SSTS, a sugar tong portion above the elbow, does not provide additional rotational stability.

## Introduction

Upper-extremity casts and splints are frequently used in the daily practice of orthopaedics and traumatology. Forearm rotation is expected to be restricted to a satisfactory level in complex injuries of the forearm, wrist, distal radioulnar joint (DRUJ), and carpal and triangular fibrocartilage. Although the traditional long arm cast (LAC) is a satisfactory method for restricting forearm rotation, it also rigidly restricts elbow movement [1]. Prolonged elbow immobilization leads to a stiff elbow [2].

In cases where rigid elbow immobilization is not essential, sugar tong splints (STSs) are commonly used for immobilization. SSTs are frequently used for treating adult and paediatric forearm fractures due to their ease of application and lack of cast- and saw-related complications [3–6]. STSs can be applied in two ways: (1) as a single sugar tong splint (SSTS) or (2) as a double sugar tong splint (DSTS) [7].

Studies have shown that SSTSs are effective for the treatment of forearm and wrist fractures [5, 7]. Moreover, DSTSs are preferred for patient groups similar to those treated with SSTSs and are successful at immobilization [6]. However, to the authors’ knowledge, no study has compared the effects of the SSTS and DSTS on restricting forearm rotation.

Therefore, the aim of the current study was to compare the effects of four different immobilization methods (SSTS, DSTS, SAC, and LAC) commonly used for restricting forearm rotation in the upper extremity. The hypothesis of the present study was that there would be no difference between the forearm rotation restriction capacities of the SSTS and DSTS.

## Methods

Local ethics committee approval was obtained for the current study (Ege University Local Ethical Committee, decision number: 24-2.1/95). The dominant, uninjured, upper extremities of forty volunteers were studied. Informed consent was obtained from all participants. Dominant extremities were used for measurements. Thirty-one of the volunteers were men, and 9 were women. The mean age was 32.48 ± 8.52 years (min.: 22; max.: 53). Thirty-six volunteers were right-hand dominant, and 4 were left-hand dominant. Forearm pronation and supination were measured without any immobilization and with use of the SAC, LAC, SSTS and DSTS. The abilities of these immobilization methods to restrict forearm rotation were compared.

### Measurements

**Fig. 1:**
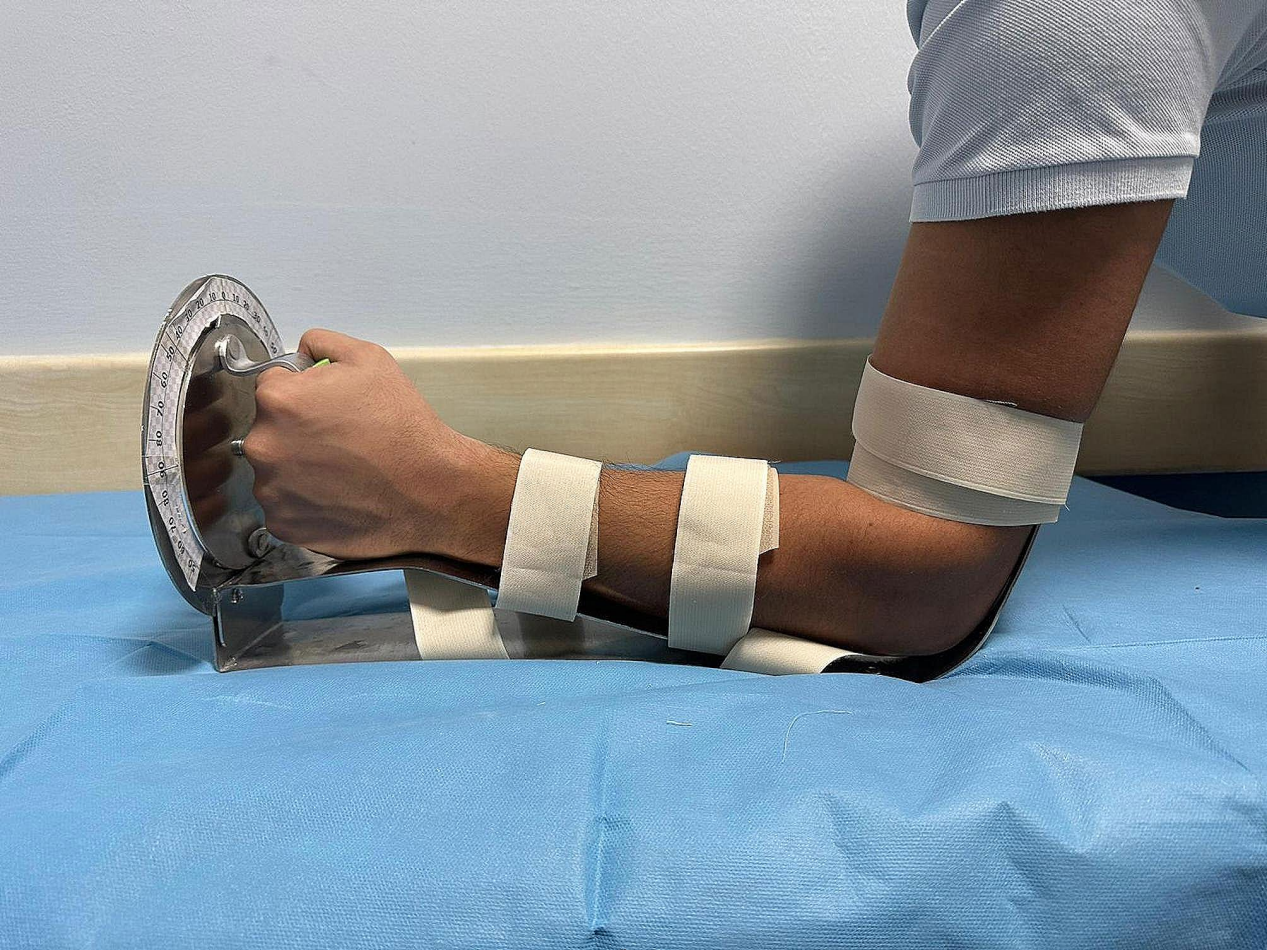
Custom-made goniometer. A circular goniometer is attached vertically to platform and grabbed by a handle. Forearm and arm fixed with help of bandages while elbow is at 90 degrees

A custom-made goniometer was used for the measurements. (Fig. 1) [1, 8]. Measurements were made under the simultaneous supervision of a hand surgeon (A.E.D.) and an orthopaedic surgeon (A.V.) with their consensus. While the volunteer was in a seated position, the extremity was placed on the goniometer and fixed with bandages to restrict arm and shoulder movement. At the starting point, the elbow was in 90° flexion, and the forearm was in neutral rotation. Basal pronation and supination of the forearm were measured, and the total rotation arc was calculated. Next, the measurements were repeated with SAC, LAC, SSTS and DSTS. Each measurement was compared to the baseline value, and the percentage of rotation restriction was noted. For example, the rotation arc of volunteer number 1, whose basal supination was measured at 90° and whose basal pronation was measured at 75°, was recorded as 165°. For the SSTA, the supination of the same volunteer was measured as 25°, the pronation was 20°, and the rotation arc was calculated as 45°. The percentage of rotation arc restriction was recorded as “(165 − 45)/165 × 100 = 73” for the SSTA. The percentage of supination restriction was “(90 − 25)/90 × 100 = 72”, and the percentage of pronation restriction was “(75 − 20)/75 × 100 = 73”.

### Preparation of the immobilization methods

Casts and splints were applied and removed by orthopaedic surgery residents under the supervision of Author 1 and Author 2. There were no splint- or cast removal-related complications.

#### Short-arm cast

One layer of circumferential cotton undercast padding was applied. A 4-inch fiberglass cast roll (Delta-Lite® Plus, BSN Medical) was used. The forearm was placed in neutral rotation during application. SACs were applied starting from just proximal to the palmar crease, then extending proximally and ending 5 cm distal to the elbow flexion crease.

#### Long-arm cast

One layer of circumferential cotton undercast padding was applied. A 4-inch fiberglass cast roll (Delta-Lite® Plus, BSN Medical) was used. The forearm was placed in neutral rotation, and the elbow was in 90° flexion during application. The LAC was applied starting from just proximal to the palmar crease, then extending proximally and ending 5 cm distal to the axilla.

#### Single sugar tong splint

A 4-inch prefabricated synthetic splint system (Dynacast® Prelude, BSN Medical), was used. The forearm was placed in neutral rotation during application. The splint was applied starting from just proximal to the palmar crease, extending proximally around the elbow and ending at the dorsal metacarpophalangeal joint. An elastic wrap was applied to fix the splint [7, 9].

#### Double sugar tong splint

A 4-inch prefabricated synthetic splint system (Dynacast® Prelude, BSN Medical), was used. The forearm was placed in neutral rotation, and the elbow was placed in 90° flexion during application. Initially, an SSTS was applied as detailed above. Then, a proximal sugar-tong part, which starts at the lateral proximal humerus level and continues around the elbow and ends at 5 cm distal to the axilla, was applied. An elastic wrap was applied to fix the splint [7, 9].

### Statistical analysis

Priori sample size calculation was performed utilizing G*Power Version 3.1.9.7 (Dusseldorf, Germany). Minimum sample size should be 31 for ANOVA: repeated measures, within factors; with an effect size of 0.25; alpha error probability of 0.05 and power of 0.95. Forty volunteers were included in the current study, power was 0.987. Data analysis was performed using SPSS Version 26 (Chicago, IL, USA). The normality of the distribution was analysed using the Shapiro–Wilk test. None of the data had a normal distribution; therefore, nonparametric tests were used. Related samples were compared using Friedman’s repeated-measures ANOVA. Wilcoxon Signed Rank test with Bonferroni correction was performed for post-hoc analysis. The level of significance was set at < 0.05.

## Results

The baseline rotation and restriction rates of the four immobilization methods are presented in Tables 1 and 2. Comparisons of the four immobilization methods are presented in Table 3. The median forearm supination, pronation and rotation arc restriction rates in the forearms with LACs were 94.67%, 99.50% and 95.56%, respectively. The limitation of forearm rotation was greatest with LACs (*p* = 0.00).

**Table 1:** Amount of forearm rotation (degrees) under different conditions

		Mean	Std. Deviation	Median	Minimum	Maximum	Range	Percentile 25	Percentile 75
without immobilization									
	supination	97,20	13,21	94,00	60,00	120,00	60,00	90,00	110,00
	pronation	66,88	13,62	70,00	40,00	90,00	50,00	60,00	75,00
	rotation arc	164,08	19,22	160,00	110,00	200,00	90,00	150,00	177,50
with SSTS									
	supination	26,03	13,57	27,50	3,00	60,00	57,00	12,50	35,00
	pronation	25,30	13,37	22,50	0,00	50,00	50,00	16,00	40,00
	rotation arc	51,33	24,20	50,00	11,00	100,00	89,00	30,00	75,00
with DSTS									
	supination	23,33	12,70	20,00	3,00	60,00	57,00	10,00	30,00
	pronation	19,28	12,41	15,00	3,00	45,00	42,00	9,00	30,00
	rotation arc	42,63	23,02	41,50	6,00	95,00	89,00	23,00	60,00
with SAC									
	supination	41,78	20,97	40,00	8,00	90,00	82,00	25,00	60,00
	pronation	23,78	11,13	21,00	5,00	60,00	55,00	20,00	30,00
	rotation arc	65,55	25,24	64,00	18,00	125,00	107,00	47,50	80,00
with LAC									
	supination	7,73	3,32	8,50	0,00	15,00	15,00	5,00	10,00
	pronation	4,10	3,40	5,00	0,00	10,00	10,00	0,50	5,00
	rotation arc	11,83	5,56	10,50	0,00	25,00	25,00	8,00	15,00

SSTS: single-sugar tong splint; DSTS: double-sugar tong splint; SAC: short arm cast; LAC: long-arm cast.

**Table 2:** Percentages of forearm rotation restrictions

	Mean	Std. Deviation	Minimum	Maximum	Percentiles		
percentage of supination restriction (%)					25th	50th (Median)	75th
SSTS	73,11	14,00	41,67	96,67	63,64	73,07	86,88
DSTS	75,87	13,12	45,45	96,47	66,67	78,37	88,73
SAC	56,31	22,48	5,26	91,11	40,91	57,33	77,09
LAC	92,01	3,32	85,88	100,00	89,17	91,49	94,67
percentage of pronation restriction (%)							
SSTS	61,77	20,35	16,67	100,00	50,00	63,40	77,78
DSTS	70,93	18,71	33,33	96,25	57,14	78,18	87,29
SAC	63,52	17,06	16,67	92,86	51,79	66,67	75,33
LAC	94,07	4,84	83,33	100,00	91,67	93,88	99,50
percentage of rotation arc restriction (%)							
SSTS	68,79	14,55	41,18	92,12	55,97	66,15	81,25
DSTS	74,04	13,79	42,86	96,36	64,11	74,73	85,79
SAC	59,53	16,62	21,88	87,14	47,80	60,56	72,43
LAC	92,85	3,11	86,11	100,00	90,63	92,72	95,56

SSTS: single-sugar tong splint; DSTS: double-sugar tong splint; SAC: short arm cast; LAC: long-arm cast.

**Table 3:** Restrictions in forearm rotation between groups

	*p* values					
	SSTS vs. DSTS	SSTS vs. SAC	SSTS vs. LAC	DSTS vs. SAC	DSTS vs. LAC	SAC vs. LAC
supination	1.00	0.25	**0.00**	**0.01**	**0.00**	**0.00**
pronation	0.18	1.00	**0.00**	0.50	**0.00**	**0.00**
rotation arc	0.50	1.00	**0.00**	**0.03**	**0.00**	**0.00**

Bold values indicate level of significance at *p* < 0.05. SSTS: single-sugar tong splint; DSTS: double-sugar tong splint; SAC: short arm cast; LAC: long-arm cast.

The median supination, pronation and rotation arc restriction rates in the SSTS were 73.07%, 63.40% and 66.15%, respectively. When the DSTS was applied, the median supination, pronation and rotation arc restriction rates were 78.37%, 78.18% and 74.73%, respectively. No statistically significant difference was detected between the SSTS and DSTS in any of the three parameters (*p* values, 1.00; 0.18; 0.50, respectively).

**Fig. 2:**
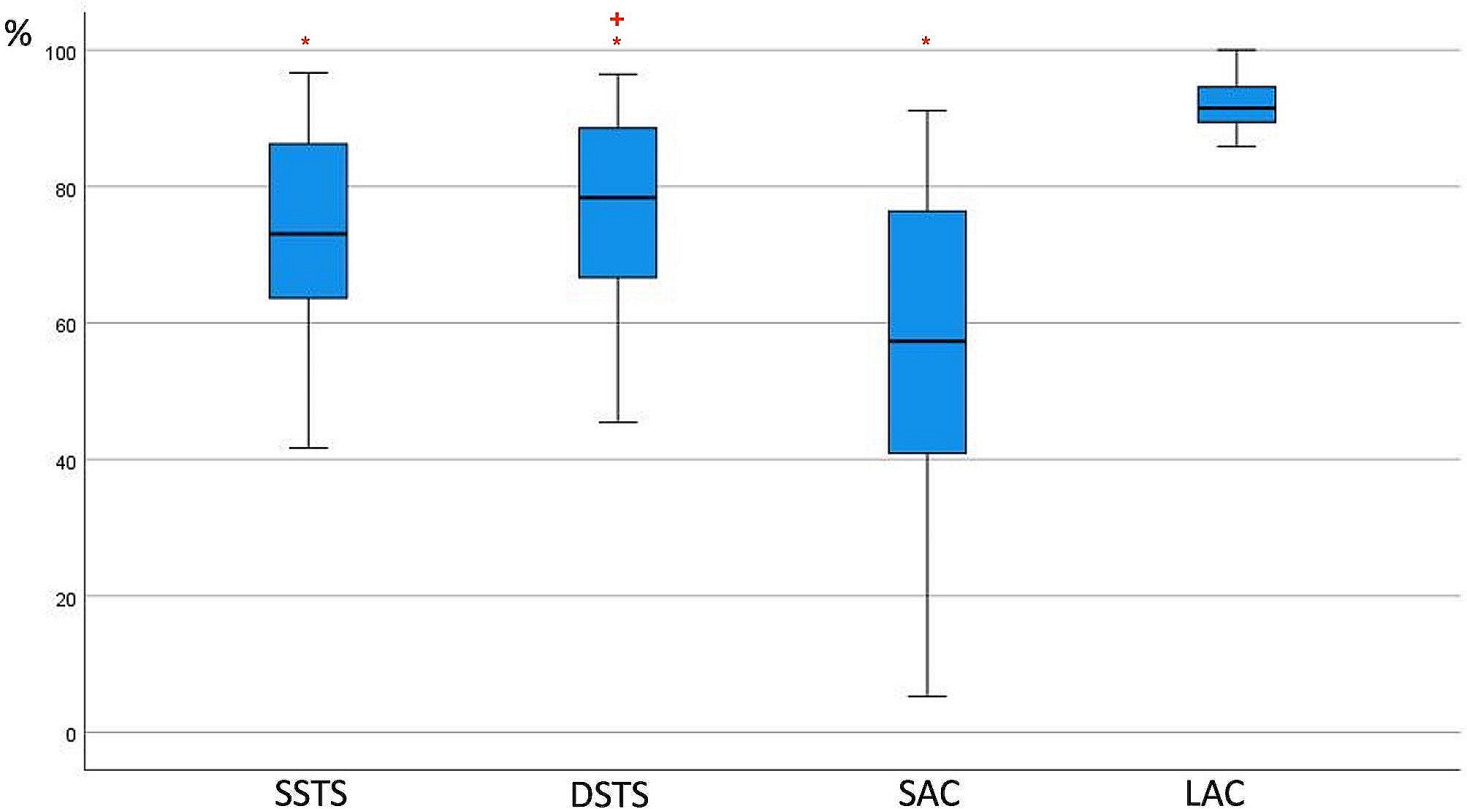
Percentage of supination restriction. Asterisks (*) indicate significant differences from the LAC, and + indicates a significant difference from the SAC. SSTS: single sugar tong splint; DSTS: double sugar tong splint; SAC: short arm cast; LAC: long-arm cast

**Fig. 3:**
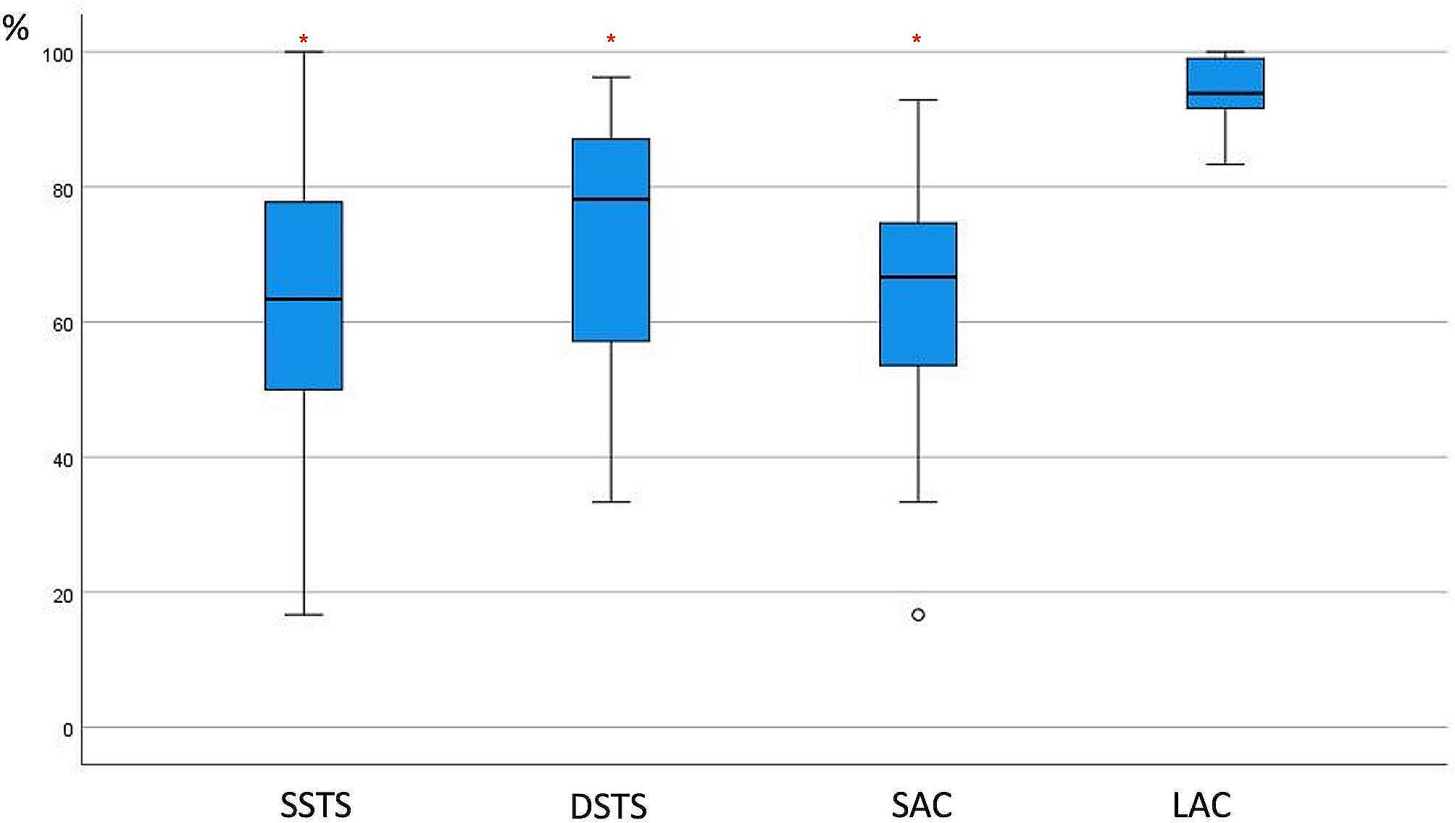
Percentage of pronation restriction. Asterisks (*) indicate significant differences from LAC. SSTS: single sugar tong splint; DSTS: double sugar tong splint; SAC: short arm cast; LAC: long-arm cast

**Fig. 4:**
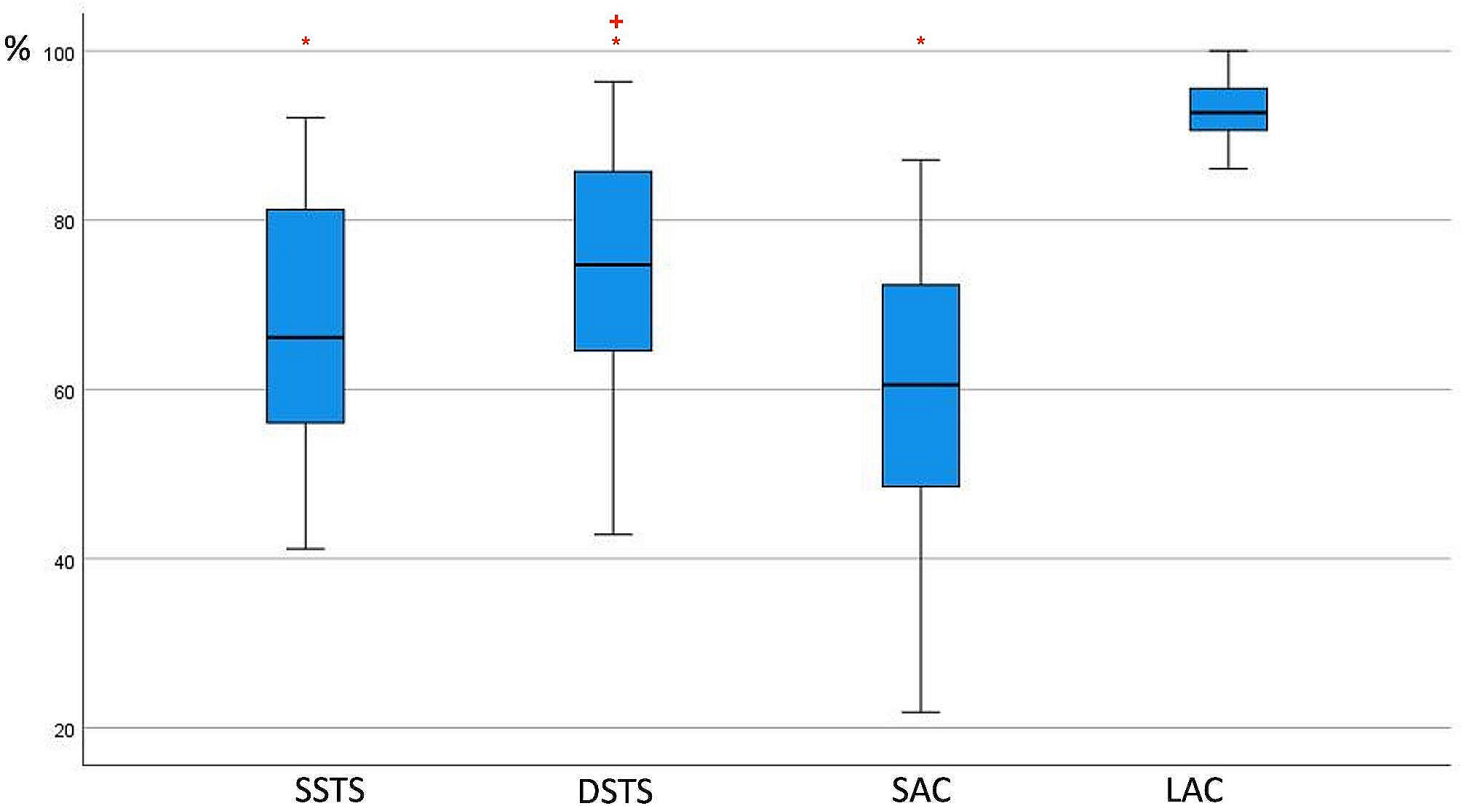
Percentage of rotation arc restriction. Asterisks (*) indicate significant differences from LAC, and + indicates a significant difference from SAC. SSTS: single sugar tong splint; DSTS: double sugar tong splint; SAC: short arm cast; LAC: long arm cast

With an applied SAC, the median supination, pronation and rotation arc restriction rates were 57.33%, 66.67% and 60.56%, respectively. Statistically significant differences were not detected between the SAC and SSTS in any of the three parameters (*p* values, 0.25; 1.00; 1.00, respectively). When the SAC and DSTS were compared, while there was no significant difference between the two methods in pronation (*p* = 0.50), a statistically significant difference was detected in supination (*p* = 0.01) or total rotation arc (*p* = 0.03) (Figs. 2, 3 and 4).

## Discussion

The current study demonstrated that the LAC is the most effective immobilization method for restricting forearm rotation, preventing more than 90% of baseline forearm motion. There is no difference between the effect of DSTS and SSTS on forearm rotation [2]. 

The SAC is an immobilization method that does not extend proximal to the elbow joint; however, the SSTS does [1]. The results of the present study confirm the findings of Kim et al., who reported no significant difference between SAC and SSTS restrictions on forearm rotation [1]. Slaughter et al. compared the effectiveness of Muenster, sugar tong, antipronation DRUJ and standard wrist splints in restricting forearm rotation [10]. They recommended the use of a sugar tong splint for maximal pronation restriction. In a recent study, Rahman et al. compared the rotational immobilization by the STS, SAC, Muenster cast (MC) and LAC [11]. They concluded that the STS yields inferior results.

The DSTS additionally includes the upper arm. For this reason, the DSTS can be expected to restrict forearm rotation better than the SSTS; however, the current study concluded that there is no difference between the two methods. Adding a splint that extends unnecessarily above the elbow will further restrict elbow movements, decrease patient comfort, and increase costs.

Rotation of the forearm depends on the harmonious functioning of the proximal and distal radioulnar joint and interosseous membrane [12, 13]. While it may be assumed that immobilization methods extending to the proximal radioulnar joint have greater capacity to restrict forearm rotation, it’s important to recognize that bones are not the sole determinants of joint movement. Pronator (pronator teres, pronator quadratus) and supinator muscles (biceps, supinator), ligaments also play role in forearm rotation [12]. The authors attempt to explain the results of the current study as follows: To limit forearm rotation, soft tissues such as skin, muscles and ligaments, as well as bony structures, must be restricted. Both SAC and LAC not only limit bony movement but also impose restrictions on surrounding soft tissues due to their complete circular envelopment. Kim et al. demonstrated in their study that increasing the length of the short-arm cast is correlated with its ability to restrict forearm rotation [8]. In this context, as expected, the most restrictions were obtained with LAC. Authors think that this is the reason why SSTS demonstrates similar efficacy to SAC despite not extending beyond the elbow. Although DSTS covers the elbow by folding around it proximally, its comparable efficacy to SSTS may be attributed to their shared characteristic of not completely surrounding the extremity.

The current study has several limitations. First, the resistance force was not standardized. Second, although all measurements were made under the simultaneous supervision of an experienced hand surgeon and an experienced orthopaedic surgeon with their consensus, rotation originating from the radiocarpal or midcarpal joints cannot be excluded. An isolated forearm rotation measurement was not possible via the current measurement method. Third, the measurements in the current study may not simulate clinical practice, as the resistance applied by a healthy extremity will not be the same as that applied by an injured extremity in a cast or splint.

## Conclusion

The LAC yields superior results in terms of restricting forearm rotation. The SAC and SSTS have similar effects on forearm rotation. The DSTS, which contains, in addition to the SSTS, a sugar tong portion above the elbow, does not provide additional rotational stability.

## Data Availability

No datasets were generated or analysed during the current study.

## Abbreviations

DSTS: double sugar tong splint
LAC: long arm cast
SAC: short arm cast
SSTS: single sugar tong splint
